# Two new Editors!

**DOI:** 10.3402/qhw.v11.31329

**Published:** 2016-02-26

**Authors:** Carina Berterö

*International Journal of Qualitative Studies on Health and Well-being* has since the first issue in 2006 developed rapidly. With the Open Access model, the journal has rapidly turned into a truly international publication. The number of submissions has increased year by year, and submissions are from a great many countries around the world. *IJQHW* is now indexed/tracked/covered in almost all relevant services, that is, PubMed, CINAHL, Scopus, etc. Even though an impact factor is not always the only sign of quality, *IJQHW* today has an impact factor of around 0.910. We are awaiting the 2015 impact factor this summer. The rejection rate is now about 50%. During 2015, the journal website received 45 278 visits by individuals in 181 countries, and approximately 360,000 full text articles were downloaded. This is a very satisfactory development and shows that the journal is immensely beneficial to both authors and readers. More and more content, the digital ease with which the content can be accessed, and targeted marketing efforts explain this positive development.

The growth of *IJQHW* has led us to enlarge the Editorial Board with several senior researchers from a variety of countries and within different disciplines, aiming to match the expectations and demands from our authors and readers alike.

In 2015, I myself was appointed Editor-in-Chief and we expanded the number of Co-Editors. Now, in early 2016, Soly Erlandsson has retired as Co-Editor after many years in this position. She will continue supporting the journal by becoming a member of the Editorial Board. I am in the process of putting together an even more international editorial team to support me in my work; Ptelene Minick will continue as Co-Editor, and Henrika Jormfeldt as well as Lisa P.L. Low have accepted to act as Co-Editors. I welcome them as competent colleagues and as my new collaborators in the work with *IJQHW*.

Henrika Jormfeldt, PhD, is an Associate Professor at Halmstad University, Sweden. She is teaching at the Postgraduate Diploma in Specialist Nursing—Mental Health Care and doing research on mental health nursing. Part of her research can be characterized as multidisciplinary. Alongside nursing, psychiatry, sociology, and social work are disciplines involved in her research. Henrika has been a registered nurse specialized in mental health nursing since 1993, and her PhD thesis, from 2007, was about Dimensions of Health among Patients in Mental Health Services. Henrika's main interests are mental health issues and inequalities in society in a broad perspective as well as research methods related to mental health and nursing—presenting a holistic view.

Lisa P.L. Low, PhD, is an Associate Professor at the School of Health Sciences, Caritas Institute of Higher Education in Hong Kong. Her research interests are gerontology and long-term care, with emphasis on the organization of care and practices, decision-making in residential care homes, discharge planning for older patients and family members, and family decision-making of older people with mild-moderate dementia using predominately qualitative research approaches. She has a deep passion and dedication to use and to continually learn to integrate qualitative research into her academic career and postdoctoral developments. This began following the completion of her Master of Philosophy (MPhil) academic degree in 1996. The MPhil thesis focused on the use of action research (combined with ethnography), and her PhD thesis in 2013 adopts a constructivist grounded theory approach. Lisa intends to continue to develop qualitative research approaches and methodologies.

Hereby, I want to thank Soly Erlandsson for contributing to maintaining the high quality of the journal for many years; I am thankful for her continuing commitment to the journal by joining the Editorial Board. I warmly welcome my colleagues Henrika Jormfeldt and Lisa P.L. Low as new Co-Editors of the *International Journal of Qualitative Studies on Health and Well-being.*

*Carina Berterö* Editor-in-Chief

